# An Update on Glioblastoma Biology, Genetics, and Current Therapies: Novel Inhibitors of the G Protein-Coupled Receptor CCR5

**DOI:** 10.3390/ijms22094464

**Published:** 2021-04-24

**Authors:** Tamara Lah Turnšek, Xuanmao Jiao, Metka Novak, Sriharsha Jammula, Gina Cicero, Anthony W. Ashton, David Joyce, Richard G. Pestell

**Affiliations:** 1 Department of Genetic Toxicology and Cancer Biology, National Institute of Biology, 1000 Ljubljana, Slovenia; metka.novak@nib.si; 2 Faculty of Chemistry and Chemical Technology, University of Ljubljana, 1000 Ljubljana, Slovenia; 3 Pennsylvania Cancer and Regenerative Medicine Research Center, Baruch S. Blumberg Institute, 3805 Old Easton Road, Doylestown, PA 18902, USA; xmjiao1@gmail.com; 4 School of Medicine, Xavier University, Santa Helenastraat #23, Oranjestad, Aruba; sriharsha.jammula@students.xusom.com (S.J.); gina.cicero@students.xusom.com (G.C.); AshtonA@mlhs.org (A.W.A.); 5 Division of Perinatal Research, Kolling Institute, Northern Sydney Local Health District, St Leonards, NSW 2065, Australia; 6 Sydney Medical School Northern, University of Sydney, Sydney, NSW 2006, Australia; 7 Lankenau Institute for Medical Research Philadelphia, 100 East Lancaster Ave., Wynnewood, PA 19069, USA; 8 Medical School, Faculty of Health and Medical Sciences, The University of Western Australia, 35 Stirling Highway, Crawley, WA 6009, Australia; JoyceD@mlhs.org; 9 The Wistar Cancer Center, Philadelphia, PA 19107, USA

**Keywords:** CCL5, CCR5, cytokines, glioblastoma, glioblastoma stem cells, intertumoral heterogeneity, perivascular niche, glioblastoma therapy

## Abstract

The mechanisms governing therapeutic resistance of the most aggressive and lethal primary brain tumor in adults, glioblastoma, have increasingly focused on tumor stem cells. These cells, protected by the periarteriolar hypoxic GSC niche, contribute to the poor efficacy of standard of care treatment of glioblastoma. Integrated proteogenomic and metabolomic analyses of glioblastoma tissues and single cells have revealed insights into the complex heterogeneity of glioblastoma and stromal cells, comprising its tumor microenvironment (TME). An additional factor, which isdriving poor therapy response is the distinct genetic drivers in each patient’s tumor, providing the rationale for a more individualized or personalized approach to treatment. We recently reported that the G protein-coupled receptor CCR5, which contributes to stem cell expansion in other cancers, is overexpressed in glioblastoma cells. Overexpression of the CCR5 ligand CCL5 (RANTES) in glioblastoma completes a potential autocrine activation loop to promote tumor proliferation and invasion. CCL5 was not expressed in glioblastoma stem cells, suggesting a need for paracrine activation of CCR5 signaling by the stromal cells. TME-associated immune cells, such as resident microglia, infiltrating macrophages, T cells, and mesenchymal stem cells, possibly release CCR5 ligands, providing heterologous signaling between stromal and glioblastoma stem cells. Herein, we review current therapies for glioblastoma, the role of CCR5 in other cancers, and the potential role for CCR5 inhibitors in the treatment of glioblastoma.

## 1. Introduction

The most aggressive and common primary brain tumor in adults, glioblastoma (GBM), is poorly responsive to therapy, including surgery followed by chemotherapy and radiation, and has one of the shortest (5 years) patient survival rates (<5%) among all cancers [1]. Successful treatment of GBM remains one of the most difficult challenges in brain cancer therapy. Trials of therapies against molecular targets that drive growth in other primary tumor types have been largely unsuccessful in GBM. The obstacles to effective therapy include therapeutic resistance of a small population of GB cells—the glioblastoma stem cells (GSCs) [2,3,4,5], significant inter- and intratumor heterogeneity, which consist of a plethora of GBM cells that differ in their genetic makeup and stage of differentiation, and the presence of a variety of stromal cells, in this cancer called GBM microenvironment (GME) [6,7]. Several comprehensive reviews have described molecular signaling pathways [8], genetics, and treatments of gliomas [9] including the repurposing of previously approved drugs [10]. The focus of this review is to extend upon recent discoveries in this rapidly evolving field.

The tumor microenvironment (TME) represents a “soil” upon which cancer cells, “the seeds,” depend [11]. In GBM, the unique stromal cell composition includes normal neuronal/glial progenitor cells, neurons, abundant resident astrocytes, endothelial cells, and a repertoire of immune-competent cells, (brain macrophage-like microglia, infiltrating macrophages, lymphocytes, and mesenchymal stem cells). Novel GBM therapies target both specific GBM mutations and the TME (stromal, and immune cells) as well as the intercellular communication, the so-called cross talk. Targeting the communication networks or nodes between the heterogeneous cells within the tumor mass that promote tumor growth and therapy resistance may be a more successful approach than targeting stromal cells directly. These “hotlines” of autocrine and paracrine information use similar “molecular hardware” (chemokines, cytokines, growth factors, enzymes, and other signal transmitters) to interconnect complex cellular activities, promoting tumor progression. Resident and infiltrating immuno-competent cells express a large number of inflammatory mediators supporting GBM aggressiveness and resistance, partially also due to GBM-derived factors that educate them to do so. Herein, we describe one tumor node consisting of the C–C chemokine receptor 5 (CCR5) and its ligands (such as CCL5) of which inhibition prevents cancer progression [12,13,14,15]. The future success of treating GBM may well depend on a better understanding of these key TME nodes that govern the highly heterogeneous and immunosuppressive microenvironment of GBM.

## 2. Glioblastoma

Gliomas are the most common primary brain tumors in adults [1]. The WHO distinguishes four grades of glioma of which GBM (WHO IV) is the most malignant. Unfortunately, GBM is also the most common glioma (5–7 cases per 100,000 individuals per year) [16]. GBM is characterized by high levels of necrosis, angiogenesis, and cellular pleomorphism. Several different approaches have been used to define the subtypes of GBM for the purpose of therapeutical stratification [17]. The classification of gliomas underwent major changes in the fourth edition of the WHO Classification of Tumors of the Central Nervous System (2016) and again by the Consortium to Inform Molecular and Practical Approaches to CNS Tumor Taxonomy (2017). These documents stratify GBM diagnosis on histology and a series of molecular markers. Glioblastoma (GBM) is now defined as a diffuse astrocytic glioma with no mutations in *IDH* genes nor *histone H3* genes [17]. The tumor is characterized by microvascular proliferation, necrosis, and/or specific molecular features (including TERT promoter mutation, *EGFR* gene amplification, and/or a +7/−10 cytogenetic signature). If one or more of these alterations is detected, the tumors are classified as “IDH-wild type GBM,” given their association with a poor prognosis even in the absence of necrosis and microvascular proliferation. On the other hand, “IDH-mutant astrocytic gliomas” comprise lower-grade astrocytoma and “grade IV astrocytoma,” which is genetically distinct from the much more common IDH-wild type “grade IV glioblastoma” in spite of similar clinical appearance. In astrocytic gliomas mutated isocitrate dehydrogenases (*IDH 1* and *2*) produce excessive amounts of oncoprotein 2-hydroxyglutarate, which leads to genomic reprogramming of cell metabolism [18,19,20]. In this review, only IDH wild-type grade IV glioblastoma and not “IDH- mutant type IV astrocytoma” will be discussed since the two grade IV malignancies differ in genetic makeup, progression rate, and disease prognosis.

### 2.1. Subtypes and Genetics and of Glioblastoma

Whether the origin of brain cells [21] or altered signaling from distinct driver mutations per se are relevant to the variety of GBM subtypes is still not clear [22,23]. In 2010, two independent studies suggested that four different genetic subtypes drive the diversity of GBMs [21,22]. Based on gene expression patterns, somatic mutations, and DNA copy number, the GBM subtypes were clustered into three major subpopulations (proneural (PN), classical (CL), and mesenchymal (MES)) guided by three key oncogenes—platelet growth factor receptor-α (PDGFRA) activation, epidermal growth factor receptor (*EGFR*) activation, and tumor suppressor neurofibromin 1 (*NF1*) deletions, respectively. The phenotype and prognosis of GBM subtypes differ. MES–GB patients have the shortest overall survival whilst the median survival of PN–GBM patients is the longest. PN–GBM tumors are characterized by high expression of OLIG2 and TCF3 genes, whereas MES–GBMs express high levels of MET, CD44, and CHI3LI/YLK40 driver genes [24], as well as a number of passenger genes (TCGA Glioblastoma Genome Atlas https://wiki.cancerimagingarchive.net/display/Public/TCGA–GBM, accessed on 22 April 2021).

Another approach distinguished among three subtypes of GBM based on phenotypes and prognosis referred to as mitotic (or favorable), intermediate, and invasive (or poor prognosis) [23]. These categories were verified by distinct genetic fingerprints showing similar but not identical trends as the TCGA-based classification [22,25]. However, the GBM subtype-specific markers are variable and simultaneously expressed across individual cells even within a single tumor [26,27]. Subtype stratification is thus confounded by tumor heterogeneity, often making targeted therapies relatively ineffective. Higher intra-tumor heterogeneity is associated with a worse prognosis of GBM patients and is a particular barrier to therapy that targets vulnerable mutations. Moreover, treatments may exacerbate poor final outcomes since irradiation [28,29] and chemotherapy alone or in combination [30] induce mutations that may augment an MES-like GBM phenotype, with greater aggression and therapy resistance.

Transcriptomic subtypes correlated with increased intratumor heterogeneity and the presence of a characteristic TME in which macrophages/microglia, CD4^+^ T lymphocytes, and neutrophils were identified by in silico analysis [31]. *NF1* deficiency resulted in MES–GBM linked to an increased tumor-associated macrophages/microglia infiltration. Short-term relapse after radiation therapy is characterized by both increased expression of MES–GBM subtype genes and a gene signature based on TME inference, associated with decreased monocyte invasion, increased macrophages/microglial cells, and CD8^+^ T cell enrichment. A recent pathogenetic characterization of GBM revealed that four immune GBM subtypes could be identified in *IDH*-wild-type GBM and *IDH*-mutant astrocytoma [25]. Taken together, higher intertumor and intratumor heterogeneity are associated with lower overall patient survival, in spite of modern modalities of standard-of-care GBM therapy [32], and novel targeted approaches are undoubtedly needed.

### 2.2. Cancer Stem Cells in Glioblastoma

The pathogenesis of GBM is initiated by transforming mutations in normal neural stem cells (NSCs), resulting in brain cancer propagating cells (BCPC) [4] that, along with the accumulation of oncogenic mutations, develop into GBM stem cells (GSC). Alternately, the oncogenic mutation in transformed NSC progenitors and fully differentiated cells, such as astrocytes, results in dedifferentiation to a GSC phenotype through enhanced expression of genes regulating “stemness” [2,22]. Similar to NSC, GSCs have the capacity for self-renewal and proliferate long term, resulting from the increased expression of stemness genes. GBMs that evolved from GSCs are the most aggressive brain tumors with an average survival of only 15 months after surgery and chemo-irradiation [1]. The slowly dividing nature, enhanced DNA repair, and multidrug resistance mechanisms [24] of GSCs promote survival after radio- and chemotherapy and facilitate tumor regrowth [5,30].

The replicative state of GSC in vivo is tightly regulated by the microenvironment (see below) of microanatomical regions within the tumor, referred to as the GSC niche [33]. The niche provides further protection of GSC from the effects of irradiation and chemotherapy. In patient-derived tissues, GSC niches are located in hypoxic regions around arterioles, wherein oxygen exchange is prevented due to tight smooth muscle cell associations with endothelial cells [34]. Moreover, GSCs are mostly localized in the subventricular zone (SVZ), the area in which normal neural stem cells reside in both fetal and adult brains [35]. However, in a cancerous brain, the microenvironment of the niche supports growth, protecting GSC from therapy and the hypoxic microenvironment maintains GSCs in a dormant state over an extended period of time [36].

High rates of therapy resistance and GBM recurrence after therapy are due to the GSC plasticity and intercellular heterogeneity resulting from GSC dedifferentiation. GSC dedifferentiation results from the loss of stemness genes by the progeny of GSCs [2,5,37]. Reciprocal signaling between GSCs and (more) differentiated GBM cells, as well as among GBMs [38], promotes malignant progression [36]. Recent single-cell sequencing studies of GBM [39] and high-throughput data analyses [7] revealed heterogeneous genetic, genomic, and epigenetic features that can only be partially explained by the tumor cell hierarchy that arises from GSCs. Moreover, GSCs reportedly transdifferente to pericytes to support GBM angiogenesis and tumor growth [40], thus to some extent also creating their own microenvironment.

Taken together intratumor cellular heterogeneity may be due to GSC cells of different brain cells and diverse dedifferentiation. Many attempts to develop targeted or tailored therapies for specific mutations or GBM subtypes largely failed due to these complexities and selective inter-GBM molecular cross talk.

### 2.3. Glioblastoma Therapy

The standard-of-care therapeutic modalities for GBM (including maximally safe surgical resection, irradiation (IR), chemotherapy with alkylating agent temozolomide (TMZ), and frequency electrotherapy of tumor treating fields (TTF) [32] has increased median patient survival from approximately16 months to 20 months after diagnosis. The high levels of GBM cell outgrowth throughout the brain make surgical resection mostly incomplete and prevent IR from being focused. Moreover, GSC plasticity, as described above, promotes therapy resistance to a much greater extent than the blood–brain barrier that is mostly compromised in glioblastoma tumors. Thus, unlike many other cancer types, in which targeted therapies have improved survival substantially, there have not been similar advances in GBM treatment [41].

Resistance of GBM is a serious problem, preventing effective treatment. The EANO and International Brain Tumor Study Group demonstrated that dose intensification fails to improve patient survival [17,42]. The combination of TMZ (standard maintenance chemotherapeutic in GBM) and IR (CATNON Trial) prolonged survival only in patients with IDH-mutant astrocytoma WHO grade 3 and not in those with GBM [43]. The prolongation of TMZ treatment from 6 to 12 cycles extended neither progression-free nor overall survival [44]. The spectra of TMZ effects on GBM cells are surprisingly poorly understood [45]. This drug acts via the electrophilic diazonium ion, which methylates guanine residues at several DNA positions, resulting in a cytostatic G_2_–M phase of cell cycle arrest and autophagy, proceeding to apoptosis depending on the cell context. TMZ also alkylates many other macromolecules, such as mitochondrial DNA and RNA as well as proteins and lipids carrying nucleophilic groups, acting at posttranscriptional/posttranslational levels [41]. The status of the enzyme methyl–guanine–methyl transferase (MGMT) counteracts TMZ activity and is a criterion for TMZ use. However, MGMT may not be a useful predictor of TMZ activity [45]. Unfolded protein responses (UPRs) in the endoplasmic reticulum (ER) cause “ER stress” (ERS) and are also tightly connected processes that determine cell function, fate, and survival of cancer cells. Changes in ER–UPR mechanism can govern multiple protumoral attributes in cancer, as extensively reviewed recently [46]. ER stress–UPRs are involved in the pathophysiology of GBM through PERK pathway regulation of GSC self-renewal and differentiation and therefore provide novel therapeutic targets [47]. ER–UPR pathway also regulates resistance to chemotherapy by also involving ATF6 and IRE1α proteins [48] and thereby controls the tumor–microenvironment cross talk in GBM and other cancers [46].

Prolonged survival appears to require combined treatments [49]. Immunotherapies [50] are increasingly replaced by CAR-T [51] and potentially NK cell-targeted therapies [52,53]. Other approaches target tumor metabolism, such as IDH inhibitors in IDH mutated astrocytoma [9]. Novel approaches include targeting GBM-specific mutations [10] (Section 2.3.1) and repurposing drugs that target the immunosuppressive GME [54] (Section 3 and Section 4).

#### 2.3.1. Targeting Mutations in Glioblastoma

Several approaches have targeted the genetic mutations that arise in GBM. *BRAF*, which is mutated in 1 to 2% of GBM, is among the most mutated kinases in human cancer. BRAF inhibitors, such as Dabrafenib and PLX8394, have shown promise in GBM carrying mutations in the oncogene Ser/Threo-protein kinase B-Raf (proto-oncogene B-Raf) especially in cases that failed standard therapy (TMZ, carboplatin). Efficacy is enhanced further when used in combination with trametinib (selective MEK1/2 inhibitor [55].

The histone deacetylase inhibitor panobinostat and the proteasome inhibitor bortezomib synergistically induced apoptosis of adult and pediatric GBM cell lines at clinically achievable doses [56]. Further, targeting NAD^+^ biosynthesis overcomes panobinostat and bortezomib-induced GBM resistance.

The *ATRX* chromatin remodeler gene is mutated in the majority of GBMs and IDH-mutated astrocytoma [57]. The Wee1-like protein kinase (WEE1hu) inhibitor Adavosertib (ASD1775) selectively impairs the growth of *ATRX* deficient cell lines derived from GBM patients [58]. A GBM clinical trial (phase 0) demonstrated penetration of Adavosertib into CNS tumors [59], and Adavosertib is being developed for the treatment of patients with advanced solid tumors and CNS malignancies associated with genetic DNA repair mechanism deficiencies. A trial combining Adavosertib with irinotecan (Top1 inhibitor) administered orally for 5 days indicated that at the maximum tolerated dose (85 and 90 mg/m^2^, respectively) increased stable disease in children and adolescents with both solid and CNS tumors [53], suggesting further investigation is warranted.

#### 2.3.2. Metabolic Targets in Glioblastoma

Cancer cells have an unusually high mitochondrial membrane potential and, thus, retain a higher pH within the matrix. Several studies have examined the inhibition of complex I in the electron transport chain as a potential vulnerability of cancer cells [54]. GSCs preferentially use oxidative phosphorylation, while the rest of the tumor is glycolytic, suggesting a potential role for mitochondrial inhibitor therapy [60]. As discussed by Van Noorden et al. (2021) [61], GSCs reside in specific hypoxic microenvironments, or niches, where they maintained in a slowly dividing quiescent state, protecting them from the cytotoxic effects of chemotherapy and radiotherapy since these therapeutic strategies only target proliferating cells. It has become generally accepted that proliferating GBMs preferentially use aerobic glycolysis for their ATP production, whereas CSCs preferentially use OXPHOS, although due to low oxygen, anaerobic glycolysis, this would be expected; the advantage of that is these conditions keep the low levels but not excessive levels of ROS, which could be toxic. This is corroborated by the fact that CSCs need hypoxic conditions to control their stem cell fate and the low oxygen levels in the hypoxic niches limit but certainly do not eliminate the production of ATP and ROS. A similar phenomenon occurs in hematopoietic stem cells in their bone marrow niches.

A screen of low-passage sphere cultures from multiple tumors, which were pooled to establish primary high-throughput GBM sphere cells, identified a small molecule Gboxin that targeted unique features of mitochondrial pH in order to inhibit the viability of GBMs but not normal cells [62]. A stable Gboxin analog inhibited the growth of GBM allografts and patient-derived xenografts, extending the potential repertoire of new therapeutics for GBM. Metformin, which has both direct and indirect effects on CSCs and the TME, increased the progression-free survival of patients with both type 2 diabetes and GBM [63], and a combined analysis of patients in several trials (AVAglio, CENTRIC, and CORE) showed a significant hazard ratio observed for progression-free survival but not in overall survival [64].

## 3. Glioblastoma Microenvironment (GME)

### 3.1. Glioblastoma Transitions Are Affected by Stromal Cells in Therapy

The microenvironment of GBM (GME) consists of resident neurons, astrocytes, and tissue-resident macrophages, known as microglial cells, embedded in the brain-specific extracellular matrix. The GME is infiltrated by mesenchymal stem cells (MSCs), hematopoietic stem cells (HSCs), and their immuno-competent progenitors, including bone marrow macrophages, differentiated from peripheral blood monocytes and various types of lymphocytes (e.g., T cells, NK cells.) [31,65,66]. As reviewed by Broekman et al. [6], nearly all types of stromal cells cooperate with GBM cells in distinct malignant processes, e.g., proliferation, invasion, disarming immune responses, and therapy resistance. Resistant GSCs reside in a slow or nonproliferating, so-called quiescent/dormant, state in the niche. Dormancy is maintained by both intrinsic signaling, such as epigenetic modification, and metabolism and extrinsic factors provided by stromal cells. After therapy, dormant GSCs become activated, released from the niches, and may either differentiate into rapidly dividing GBM progenies or may transdifferentiate into an MES–GSC phenotype. The transition from a quiescent to a proliferative state is a dynamic and reversible process, highly regulated by stromal cell-secreted growth factors, cytokines, adhesion molecules, extracellular matrix components, as well as by exosomes and metabolites [7,28,29,30]. Even the quiescent differentiated GBM cells residing under the stressful conditions of hypoxia and an acidified milieu may re-express stemness genes [67]. Such “inverse evolution” of the CSCs from differentiated GBM cells to GSCs is explained by reversible epithelial-to-mesenchymal transition (EMT). The EMT is triggered epigenetically and is induced by transforming growth factor (TGF)-β signaling, inducing transcription factors, including TWIST, ZEB, and SNAIL, which interact in different combinations with specific miRNA200 and miRNA34 [68]. The emerging concept from numerous studies is that the stepwise EMT program generates various intermediate metastable states, hybrid epithelial–mesenchymal phenotypes that express stemness-related genes and pathways before acquiring full mesenchymal traits [69]. These GBM–GSC states, in addition to conveying enhanced invasion, also show resistance to apoptosis. Recently Smigiel et al. [70] revealed that senescent premalignant breast carcinoma epithelial cells (Es) could be triggered by stromal cells to undergo EMT transition, with intermediate breast cancer stem cells showing a mesenchymal and highly invasive breast stem cell phenotype. In GBM, IR and chemotherapy induce cell-cycle arrest, resulting in senescent- domant cells. 

Since several studies confirmed that GBM therapy results in recurrences with more invasive mesenchymal subtypes, it has been hypothesized that this process involves a proneural (PN) to mesenchymal (MES) GSC subtype reprogramming, or “PMT transition” [22,24,25].

### 3.2. The Immune Glioblastoma Subtypes and Immunotherapy

The cellular composition of the GME varies by tumor subtype. The MES subtype microenvironment includes endothelial cell markers and enrichment of macrophages, microglia, CD4 T cells, and neutrophils, but with a decrease in activated cytotoxic natural killer (NK) cells [31,66,71]. An increase in macrophages and microglial cells are found in the TME of early GBM recurrences, consistent with a model in which the GME regulates GBM cellular phenotype and drives GSC plasticity [72] and therapy-resistant tumors [73] (reviewed by Chen et al. [74]). New light has been shed upon the MES–GBM subtype through comprehensive genomic and phosphor–proteomic analyses of 99 GBM samples, which showed upregulation of hypoxia and angiogenesis pathways [25], together with activation and polarization to the M2 macrophage phenotype. snRNA-seq data identified mesenchymal features in both tumor and immune cells. In one of the four GBM immuno-subtypes, EMT-related genes were expressed by nontumor cell types, including the tumor-associated macrophages, T cells, pericytes, endothelial cells, and oligodendrocytes [69].

It is believed that GME stromal cells participate in a two-way dialogue with the GBM cells, promoting an immunosuppressive GME [75], including tumor-infiltrating immune T regulatory cells (Tregs), MSCs, together with decreased expression of costimulatory molecules and the secreted immunosuppressive factors, such as TGF-β, interleukins (IL-10 IL-6), and indoleamine 2,3-dioxygenase (IDO), which inhibits immune effector activation and prevents antitumor immune responses [76,77]. These heterotypic cellular interactions, which contribute to tumor progression and resistance [34,38,78,79], are maintained through the release of cytokines, extracellular vesicles, nanotubes, and microtubules [80], together with direct cell–cell contact [81].

Several mechanisms contribute to the immunosuppressive GME. 11% of recurrent tumors harbor mutations in the immunosuppressive protein latent TGF-β and its cognate-binding protein *LTBP4* [30]. TGF-β promotes the invasive mesenchymal GBM phenotype while silencing of *LTBP4* suppresses TGF-β activity and decreased cell proliferation. Firstly, in recurrent GBMs (wild-type *IDH1*), high *LTBP4* expression is associated with a worse prognosis, suggesting the TGF-β pathway is a potential therapeutic target for GBM. Secondly, the expression of immune checkpoint proteins, which mediate the direct contact between GBM and cytotoxic CD8^+^ T cells, such as the programmed cell death protein 1 (PD-1) and its ligand (PD-L1) and cytotoxic T-lymphocyte protein 4 (CTLA-4), can be inhibited by targeted antibodies. Phase II and III clinical trials are currently assessing anti-hPD-1 and anti-CTLA-4 antibody-based drugs in GBM [82] (see Section 5.2).

## 4. Cellular Cross Talks by Chemokines and Their Receptors

Chemokines are chemotactic cytokines that cause directed migration of stromal cells, such as leukocytes [83]. Chemokines, of which there are 48 in total, are low molecular weight proteins, from 8 to 14 kilodaltons (8–14 kDa), which are divided, based on the location of the two cysteine residues located at the amino terminus, into four families: CXC chemokines, CC chemokines, C chemokines, and CX_3_C chemokines [84]. In summary, 19 unique G protein-coupled receptors (GPCRs) interact with the 48 distinct chemokines, and the resulting signaling alters transcription of target genes, regulating cell motility and invasion, interactions with the extracellular matrix, and cell survival. Chemokine signaling coordinates cell movement during inflammation, as well as the homeostatic transport of hematopoietic and mesenchymal stem cells, myeloid cells, lymphocytes, dendritic cells neutrophils, and cancer-associated fibroblasts [83].

In cancer, chemoattractions among cancer and immune cells are, to a large extent, mediated by chemokines and their receptors [52,78]. In GBM, the receptors CXCL12, CXCR4, and CXCR7 maintain the interaction between endothelial cells and GBM, promoting angiogenesis and homing GSCs to their perivascular niches [34,85,86]. Additional roles have been ascribed to CCR5 as well as CCR3 and CCR1 [87,88]. CCL5–CCR5 signaling favors malignancy by both direct effects and indirect effects on the GME recruiting immunosuppressive leukocytes, lymphocytes, and macrophages [89,90]. GB-associated macrophages (TAMs; microglia and infiltrating monocytes) undergo polarization into either M1 proinflammatory or M2a/b/c anti-inflammatory macrophages [91]. Gene expression analysis elucidated that among TAMs, microglia express primarily proinflammatory cytokines and chemokines, whereas infiltrating macrophages express predominantly anti-inflammatory or immunosuppressive cytokines [90].

### 4.1. Chemokine Receptor CCR5 and the LIGAND CCL5 in Cancers

The seven transmembrane G-protein coupled C–C chemokine receptor type 5 (CCR5) binds multiple ligands, including C–C motif chemokine 3 (CCL3/MIP-1α); C–C motif chemokine 3-like 1 [LD78-beta(1–70)]; C–C motif chemokine 4-like (CCL4/MIP-1-β); C–C motif chemokine 5 (CCL5); C–C motif chemokine 8 (CCL8/MCP-2); Eotaxin, alternatively known as (CCL11); C–C motif chemokine 13 (CCL13/MCP-4); and C–C motif chemokine 16 (CCL16/HCC-4). Upon binding of its ligand, the cognate GPCR undergoes a conformational change. This causes the dissociation of the Gαi and the Gβγ subunits and the induction of downstream signaling. The Gβγ subunit activates phospholipase Cγ to cause the hydrolysis of phosphatidylinositol 4,5-bisphosphate (PIP_2_) into both inositol triphosphate (IP_3_) and diacylglycerol (DAG). The IP_3_ causes a rapid increase in cytosolic Ca^+2^ cations. Adenyl cyclase is activated by Gαi. The CCR5 activation of Ca^+2^ signaling and cellular migration is preserved in both immune [84] and cancer cells [92,93]. Additional pathways induced by CCR5 include the protein 3-phosphoinositide-dependent protein kinase-1, PDK1, crucial for activating PI3K–Akt kinase pathway that signals via different transcription factors to induce cell survival, glycolysis, cell proliferation, immune cell differentiation, growth, and proliferation of both differentiated GBM and GSCs [13,15,84].

The role of CCR5 in the growth, invasion, and metastasis of cancer has been extensively investigated. CCR5 is overexpressed breast [84,94], prostate [92], pancreatic, colorectal [95,96], head and neck [97], gastric [87], and esophageal [98] cancer as well as acute lymphocytic leukemia [99], melanoma, Hodgkin’s lymphoma, and other tumors [88,93]. In humans, CCR5 has the peculiar characteristic of being a developmentally nonessential gene, which participates in diverse pathological processes, including infection with HIV [100,101,102,103], progression of stroke [104], and cancer metastasis [84,93,105]. Although metastasis involves multiple distinct steps, CCR5 was shown essential to govern the homing step of breast cancer metastasis in mice, as demonstrated by the anti-metastatic activity of the CCR5 inhibitor maraviroc [84,93,105]. Subsequent studies showed the importance of CCR5 in both the formation of new metastasis and the growth of previously formed breast [105] and gastric cancers [106].

The chemokine CCL5, previously known as T-cell-specific protein RANTES-(regulated on activation, normal T-cell expressed, and secreted), is a CC chemokine ligand, both known for its role in inflammatory diseases and in cancer progression [15,107]. The chemokine CCL5 also binds to C–C chemokine receptor type 1 (CCR1) and type 3 (CCR3), CCR5, and to the probable G-protein-coupled receptor 75. The signaling between CCR5 and other chemokines acts via calcium signaling [13,93,108].

### 4.2. Network of Receptor CCR5 and CCL5 Interactions in Glioblastoma

A complex network of interactions occurs between CCL5 and CCR5 in GBM including GBM cells, GSCs, and the GME cellular components of the tumor tissue (reviewed by [15]). In early studies, tumor tissues from patients with several types of primary gliomas showed increased expression of the ligand CCL3L1, together with the receptors CCR3 and CCR5. Overexpression of CCL3L1 in a GBM cell line enhanced tumor cell proliferation [109], suggesting that enhanced autocrine CCR5 signaling promotes GBM growth. CCL5 (and CCL2) mRNA and protein are elevated in both serum and tissues of GBM patients thought to be derived from immune cells including macrophages [110]. Hypoxia and macrophages were shown to promote GBM cell invasion via CCL4–CCR5 [111]. High levels of CCR5, C–X–C chemokine receptor type 4 (CXC-R4), atypical chemokine receptor 3 (CXC-R7), C–C chemokine receptor type 7 (CCR-7), and C–C chemokine receptor type 10 (CCR-10) were linked to poor prognosis GBM [112]. In primary GBM, increased expression of CCR3 and CCR5 correlated with increased activated p-Akt correlating with earlier relapses and shorter overall patient survival [14]. Levels of CCL5 and CCR5 expression correlated with poor prognosis and average survival time of GBM patients [89].

**Figure 1:**
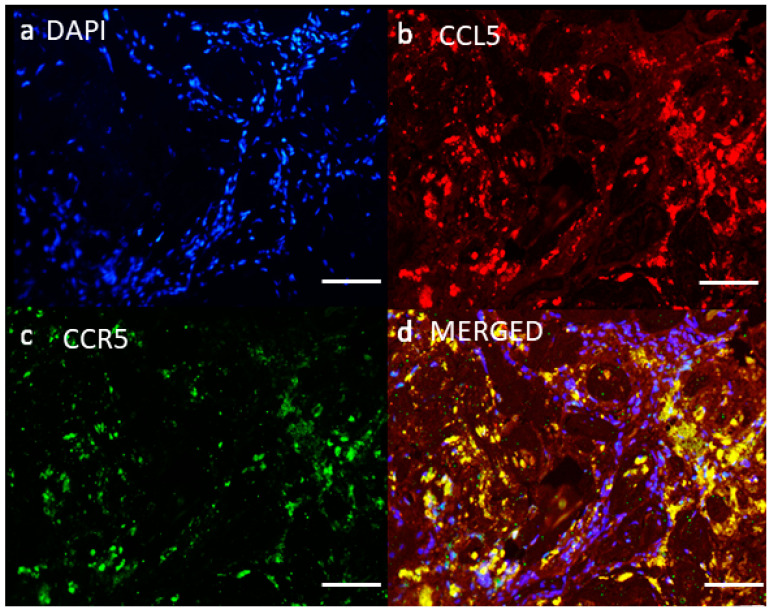
CCL5 and CCR5 are expressed in glioblastoma tissue. Fluorescence immunohistochemical staining of CCL5 and CCR5 antigen was performed on the glioblastoma section. (**a**) Nuclei were stained with DAPI (blue), (**b**) CCL5 with Alexa Fluor 546 (red), and (**c**) CCR5 with Alexa Fluor 488 (green) dye. (**d**) Merged images represent colocalization (yellow color) of CCL5 and CCR5. Microscopy was carried out at 20× objective magnification. Scale bar represents 100 µm.

Thus, CCR5 signaling can favor cancer progression either directly by affecting proliferation, cell survival, and migration of cancer cells, or indirectly, by recruiting protumor and/or anti-inflammatory effector cells [15]. GBM cells express both CCR5 and its ligand CCL5 promoting cell-autonomous signaling [13]. By the same token, GSC may also interact with differentiated GBM cells, as Wang et al. [36] demonstrated that differentiated tumor cells promote the glioblastoma hierarchy and tumor growth through a paracrine feedback loop, describing neurotrophin signaling in cooperation with stem cell-like tumor cells. We confirmed that CCL5 and CCR5 proteins were overexpressed in glioma tissues (Figure 1, unpublished), and in a cohort of 65 patients, also mRNA levels were significantly higher compared with their normal counterparts. The abundance of CCL5 and CCR5 increased with the stage of the disease. In isolated primary GSCs, only CCR5 was detected, suggesting paracrine interactions among GSCs and stromal cells. CCL5 autocrine signaling in high-grade glioma promoted growth regulation via the mesenchymal (MES) GBM–GSC subtype, which also expresses higher levels of the stemness marker CD44, a nonconventional or “auxiliary receptor” for CCL5 [113]. The MES–GBM molecular subtype is characterized by loss of the tumor suppressor gene neurofibromatosis type 1 (*NF1*) that negatively regulates CCL5 expression through suppression of AKT/mTOR signaling. In mice, CCL5 knockdown in MES–GBM cells reduced tumor cell survival in vitro and increased host survival in an in vivo tumor model [114]. Studies exploring the gene expression distribution of CCR5 and CCL5 among different genetic GBM subtypes showed the highest CCR5 levels in the CL–GBM subtype and the lowest in MES–GBM subtype, wherein CD44-mediated signaling may be predominant [13]. CCR5 plays a significant role in three major processes promoting GBM progression through expression on GSCs, GBM, and cells within the GME. Since maraviroc significantly inhibited CCL5 and MSC cells induced GBM and GSC invasion, we proposed targeting the CCL5–CCR5 signaling axis as novel glioblastoma therapeutics [115].

## 5. Targeting Chemokine–Receptor Axis

### 5.1. Targeting Glioblastoma Microenvironment and GSC Niches

Recent studies have begun to dissect the role of distinct cell types that govern CCR5-CCL5 autocrine and paracrine signaling in GBM progression [116]. CCR5 governs several different cell types that contribute to the progression of GBM and GSCs homing their niches. Within the niche, chemoattraction between dormant GCS and arteriole endothelial cells is dependent on CXCR12 (SDF-1α):CXCR4. We showed that lysosomal cathepsin K, by specific proteolytic cleavage at the *N*-terminus of the chemokine CXCR12, disrupted CXCR12:CXCR4 mediated attraction of GSC to the niche endothelium, enabling their release from the niche [33,85,86]. By fluorescent immunohistochemistry, we have shown stromal cells, including macrophages, microglia, and mesenchymal stem cells, associate with GSCs in the vicinity of the niche [34]. Tumor-associated type 2 M macrophages (TAM)-mediated immunosuppression also involves CCL5–CCR5. Overall, the pharmacological blockade of CCR5 prevents the appearance of immunosuppressive M2 macrophage and microglia phenotype as evidenced by the loss of M2-macrophage markers ARG-1 and IL-10 [84,95]. The loss of M2-polarisation correlated with a significant decrease in microglia migration, due to the inhibition of the PI3–AKT pathway. Moreover, under conditions mimicking highly malignant GBM, CCR5 blockade induced an M1 phenotype associated with increased antitumor properties and a reduction in tumor growth [15]. It is thus no surprise that levels of CCL5 and CCR5 expression correlated with poor prognosis and average survival time of GBM patients [89].

**Figure 2:**
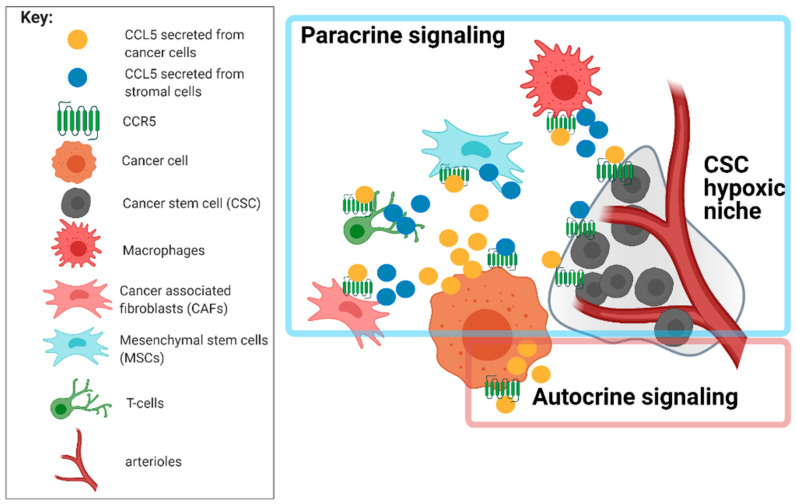
CCL5–CCR5 signaling in cancer microenvironment (created with BioRender.com). Various cell types in the GBM tumor microenvironment express both CCL5 and its receptor CCR5, contributing to cancer progression and cancer stem cell homing to their niche. GBM cells expressing CCR5 can be activated by stromal cells secreting CCL5 in paracrine signaling. Stromal cells expressing CCR5 can be activated by cancer cells secreting CCL5. In high-grade gliomas, cancer cells may establish an autocrine chemokine stimulation, becoming independent of the stroma.

The communication of GBM with immunocompetent cells follows two pathways, either (a) external activation of glioblastoma cells expressing CCR5 or (b) GBM cells expressing CCL5 activate stromal cells [15]. In most commonly observed situations, the host immune cells secrete CCL5, activating CCR5 expressed on GBM cells [111]. CCL5 in macrophage-conditioned media enhanced GBM cell invasion under hypoxic conditions, and CCL5 was critical for Neurofibromatosis-1 glioma growth in response to tumor-associated microglia [117]. CCL5 neutralizing antibodies reduced glioma tumor growth in a murine model. Secretion of CCL5 by GBM cells also affects stromal cells that express CCR5, resulting in an immunosuppressive GME. In the GME both M2 macrophages, and T-reg lymphocytes express CCR5 and are recruited to the GME via CCL5–CCR5 [105]. These results confirm the early hypothesis [109] that the overexpression of CCR5 ligands by GBM cells attracts effector cells to modulate local immunity (Figure 2).

### 5.2. CCR5 (CCR5i) Inhibitors as Adjuvant Therapy

CCR5 antagonists (such as the small molecular inhibitor maraviroc and the humanized monoclonal antibody leronlimab) inhibit HIV-1 viral entry into T cells via binding as an allosteric inverse agonist, locking CCR5 in an inactive conformation [118]. These compounds are now being repositioned to target CCR5 in cancer with active clinical trials for colon cancer and metastatic breast cancer [12,105].

One common mechanism of action for many chemotherapeutics involves the DNA damage repair response, and the potential synergy of CCR5 inhibitors with these agents is being examined at present. CCR5 augments DNA repair and is re-expressed selectively on cancerous, but not normal, breast epithelial cells. CCR5 inhibitors may therefore enhance the tumor-specific activities of DNA damage response-based treatments and allow a dose reduction of standard chemotherapy and radiation. Recent studies have shown functional synergy between the DNA damaging agent, doxorubicin, and the CCR5 inhibitor maraviroc [87,88,92,95,99,119]. DNA damage repair pathways also contribute to the therapeutic resistance of GSCs [2], and therefore, maraviroc and leronlimab are good candidates for adjuvant GBM therapy.

GBMs are cancers with a hypermutated phenotype that may benefit from immune checkpoint inhibitors because of increased neoantigen load [75,120,121]. Tumors evade immune destruction by actively inducing immune tolerance through the recruitment of Tregs, which are T cell surface glycoprotein CD4 positive (CD4^+^), interleukin-2 receptor subunit alpha positive (CD25^+^), and forkhead box protein P3 positive (scurfin) cells. The recruitment of immune cells, such as (tumor-infiltrating) lymphocyte (TIL), the myeloid-derived suppressor cells (MDSCs), (tumor-infiltrating) monocytes-macrophages (TAMs), innate lymphoid cells (ILCs), regulatory T cells (Tregs) [122], multipotent MSCs, immature dendritic cells (DCs), and NK cells [52] contribute to tumor-induced immunosuppression [123].

Evidence suggests CCR5 and its ligands participate in the immune checkpoint response. Programmed cell death protein 1 (PD-1) signaling is an important mechanism by which tumors escape antitumor immune responses. Tumor-infiltrating lymphocytes are an important biomarker for predicting responses to PD-L1 blockade therapy. Analysis of responses to CTLA-4 and PD-1 antagonists revealed that tumors responsive to these immunotherapies tend to be infiltrated with T cells, referred to as a “T cell-inflamed” TME [124,125,126]. Expression of the *CCL5* gene was upregulated in PD-L1-positive melanoma tumors, along with interferon gamma (IFN-γ) and several IFN-γ-regulated genes [127]. Tumor mutational burden and a T-cell-inflamed gene expression profiles were independently predictive of response to the PD-1 antibody pembrolizumab [126]; high concentrations of the CCR5 ligand’s CCL3 and MIP-1-β, seen in pretreatment tumor specimens, were associated with worse patient overall survival after anti-CTLA-4 and carboplatin and paclitaxel treatment was given for melanoma [126]. The potential for synergy between CCR5 inhibitors and the canonical immune checkpoint inhibitors is being explored through clinical trials of Pfizer and Merck. In these current trials, CCR5 inhibitors are combined with the checkpoint inhibitor, pembrolizumab, for the treatment of metastatic colon cancer (NCT03631407, NCT03274804). The potential role for CCR5i in augmenting immune checkpoint therapy for GBM remains a subject of considerable interest.

## 6. Conclusions

Glioblastoma is the most frequent, aggressive primary brain tumor in humans, in which standard-of-care therapy has not significantly improved patients’ survival over the past decade, emphasizing the need for novel therapeutic approaches. The challenge to improving survival in GBM include the presence of glioblastoma stem cells that both resist irradiation and chemotherapeutics and evade immune cell-killing mechanisms by altering their genotype via endogenous genomic plasticity. Herein we have emphasized the mechanisms of GBM therapy resistance are due to collaborative interactions, between GBM, GSC, and GME involving networks of immune cells, monocytes, macrophages, microglia, and mesenchymal stem cells. Rather than targeting the stromal cells in the glioblastoma environment, we believe that inhibiting cross talk within the GBM TME may be a more efficient way to disable tumor proliferation. In this review, we have focused on one communication axis mediated by CCR5–CCL5 signaling among glioblastoma GBM–GSC. CCR5 is expressed on several cell types within the GBM TME and we have outlined compelling evidence for CCL5–CCR5 in invasive and metastatic behavior of many cancer types, known to participate in driving tumor progression, invasion, and metastasis. High expression of CCR5 and CCL5 in glioblastoma tissue is associated with poor prognosis of patients. Autocrine and multicellular paracrine CCL5–CCR interactions represent a new node of cross talk that should be considered as a target for eliminating GBM and GSC activation. Clinical trials have recently opened targeting CCR5 using a humanized monoclonal antibody (leronlimab) for metastatic triple-negative breast cancer (TNBC) NCT03838367 or a small molecule inhibitor maraviroc for metastatic colon cancer (NCT03631407, NCT03274804). Based on previous studies of GBM, consideration of clinical trials with CCR5 inhibitors for GBM warrant further consideration.
